# Volcanic contribution to the 1990s North Pacific climate shift in winter

**DOI:** 10.1038/s41598-023-32956-z

**Published:** 2023-04-06

**Authors:** Chi-Hua Wu, Shih-Yu Lee, I-Chun Tsai, Chein-Jung Shiu, Yi-Ying Chen

**Affiliations:** Research Center for Environmental Changes, Academia Sinica, 128 Academia Road, Section 2, Nankang, Taipei, 115 Taiwan

**Keywords:** Atmospheric science, Climate change

## Abstract

It is debatable whether external forcing can change the state of the climate. By investigating decadal changes with and without including the 1990s stratospheric volcanic aerosols, we explored the volcanic eruptions contribution to decadal climate regime shifts occurring in boreal winter over the North Pacific. The volcanic eruptions contribution can be characterized as a series of rapid changes, including the strengthening and poleward shift of the midlatitude westerly jet stream. In addition to the short-lived radiative effects primarily induced by the 1991 Mount Pinatubo eruption, the volcanically driven decadal change can be observed in the mid-to-late 1990s, suggesting a time-lagged characteristic of the volcanic climate impact. Compared with the decadal change irrelevant to volcanic eruption, the decadal state more dramatically enters into the next phase when volcanic forcing is included. The climate oscillation-related pattern shifts that occurred across the 1990s can provide insights into volcanically induced changes in decadal atmospheric circulation.

## Introduction

Strong volcanic eruptions substantially affect the Earth’s climate, causing concerns related to atmospheric dynamics, radiative effect, and chemical composition^1,2^. Perturbations occur in climate variability modes, such as the Arctic Oscillation (AO)^3^ and El Niño/Southern Oscillation^4,5^, in the few years immediately after large eruptions. The climate perturbation may be short range, followed by changes in the Intertropical Convergence Zone^6^ and monsoon^7,8^, and the perturbation can dynamically control global post-eruption pattern shifts in temperature and precipitation^9–11^. However, the investigation of volcanic effects in a longer range, such as the decadal time-scale, remains challenging. Whether the volcanically induced change is a modulator and driver of climate variability remains controversial^12^; this represents a major challenge in temperature reconstruction and future projection.

The volcanic contribution to climate change^13^ may provide insights into the origins of multi-decadal to centennial climate variability. Regarding deglaciation in the early Holocene, a higher frequency of volcanic eruptions might have altered (paleo)climate as a cluster forcing^14,15^. The volcanically related mechanism could force the initiation of centennial cooling and sea ice expansion^16^, particularly the initiation of a returned cold condition of the Little Ice Age through the mid-nineteenth century^17,18^. Climate modeling studies have suggested that climate models that do not include volcanic effects overestimate the tropospheric warming over the historical period^19^. The volcanically induced changes can have competitive effects to the effects of climate change driven by other external forcings.

Studies have considered the volcanic eruptions contribution to the decadal change in the sea level and ocean heat content^20,21^. The western tropical Pacific sea level shift since the early 1990s was attributed to the strengthening of trade winds^22^, differing from the paradigm that the Pacific decadal variability can be driven by oceanic forcing. Changes in tropospheric circulation occurred worldwide in the 1990s. Along with the changes occurring in the 1990s, the decadal change in winter^23^ strengthened anticyclonic conditions in the lower troposphere (directly corresponding to increased trade winds) and resulted in a northward shift of the midlatitude westerly jet stream, which corresponded to a weakened Arctic vortex-like circulation. This study examined whether the decadal change in the upper-tropospheric circulation in the Northern Hemisphere is volcanically driven, particularly in terms of Pinatubo’s contribution^19,24,25^ on a decadal or longer scale and the associated physical mechanism over the North Pacific. This paper is organized as follows. “Materials” Section describes the materials including multiple reanalysis datasets, the decadal characteristics of volcanic eruptions, climate experimental simulations, and focused climate variability mode indices. In “Results” Section, the volcanic contribution to the decadal change that occurred in the 1990s is explored, with a focus on the North Pacific tropospheric circulation in winter. The findings are discussed and summarized in “Discussion” and “Conclusion” Sections.

## Materials

The observed dynamic fields from seven reanalysis datasets were analyzed, namely the Modern-Era Retrospective analysis for Research and Applications version 2 (MERRA2) of National Aeronautics and Space Administration (NASA)^26^, the European Centre for Medium Range Weather Forecasts (ECMWF) Reanalysis version 5 (ERA5)^27^, the Japanese 55-year Reanalysis (JRA55)^28^, the National Centers for Environmental Prediction (NCEP)/National Center for Atmospheric Research (NCAR) Reanalysis 1 (NCEP R1)^29^, the NCEP Climate Forecast System Reanalysis (CFSR)^30^, the National Oceanic and Atmospheric Administration (NOAA)-Cooperative Institute for Research In Environmental Sciences (CIRES)-Department of Energy (DOE) 20th Century Reanalysis version 3 (20CRv3)^31^, and the ECMWF Atmospheric Reanalysis of the 20th Century (ERA20C)^32^. The spatial resolution of the data ranged from 0.25° to 2.5° in terms of the longitude/latitude and from 42 to 17 pressure levels. The seasonal mean in December to February was averaged from daily or higher temporal resolution data obtained from the datasets; the year is identified for December (e.g., December 1995–February 1996 for the 1995 winter). Diagnostic analyses were performed using reanalysis data individually and in combination (i.e., as ensemble mean at a horizontal resolution of 2.5°).

Historical volcanic events and the associated volcanic explosivity index (VEI)^33^ were obtained from the Global Volcanism Program of National Museum of Natural History (Smithsonian Institution, https://volcano.si.edu/). Climate modeling results obtained from the literature^34^ and the corresponding data archives^35^ were explored in this study. We investigated two climate simulations that were conducted using the Norwegian Earth System Model (NorESM) with and without historical volcanic forcing (60 ensemble members for each). The volcanic forcing was implemented by scaling the shape functions with aerosol loading from the ice-core-based reconstruction. Control (experimental) runs with (zero) volcanic forcing spanned the years 1980–2005 (1990–2004). We referred to the studies conducted by Bethke et al.^34^ and^36^ for the experimental design and detailed description of volcano-related model outputs.

We explored the temporal evolution of sea level pressure and atmospheric dynamic fields by performing an empirical orthogonal function (EOF) analysis. To evaluate the decadal change, we applied a 7-year running mean before performing the EOF analysis. Moreover, we examined the difference between the periods 1996–2001 and 1990–1995; testing the difference can provide the confidence level of the decadal change. We analyzed the characteristics of AO, Pacific decadal oscillation (PDO), and North Pacific gyre oscillation (NPGO). We downloaded the AO index for the period from 1950 to the present, the PDO index for the period from 1900 to the present, and the NPGO index for the period from 1950 to the present.

## Results

**Figure 1:**
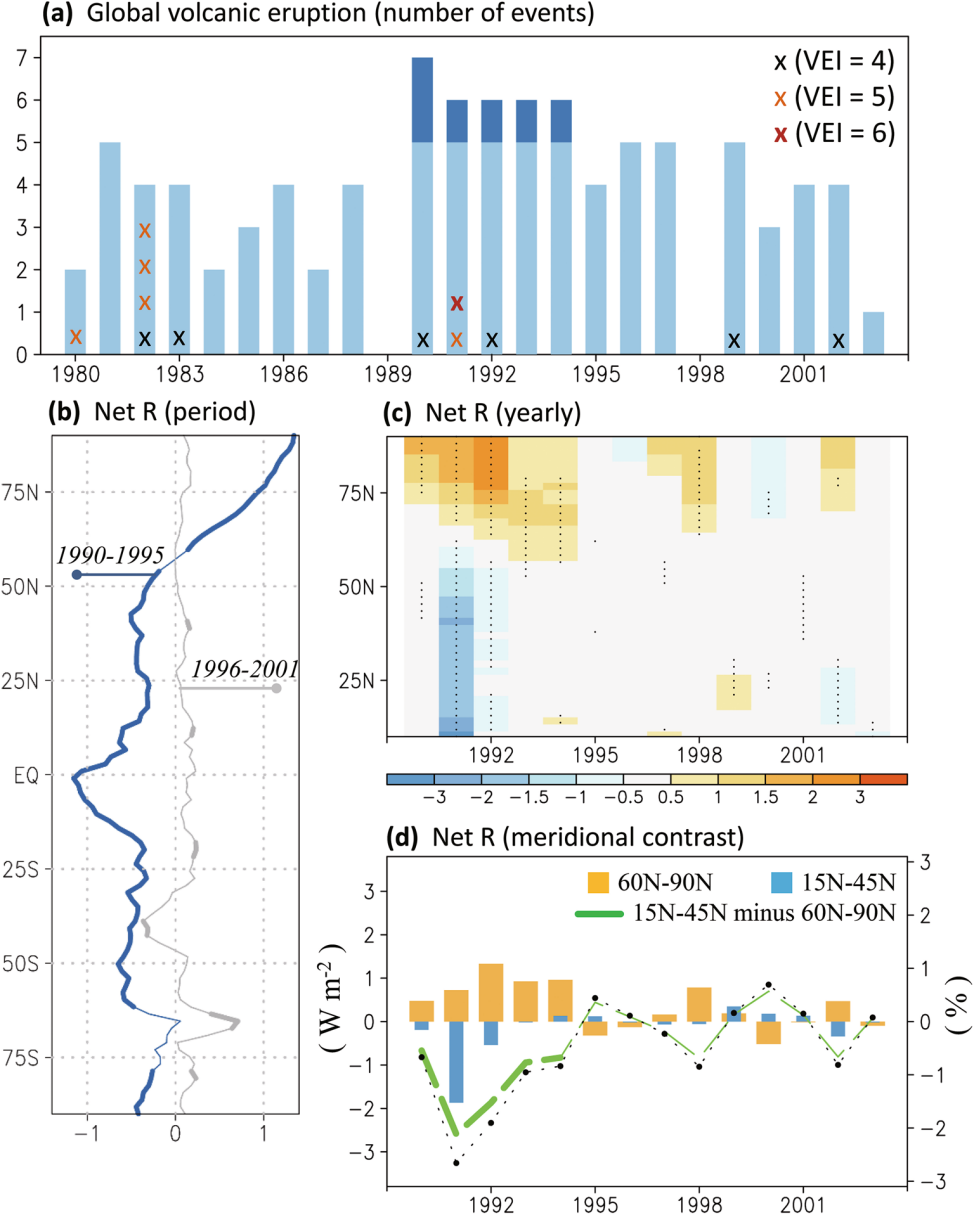
Radiative effects of decadal volcanic eruptions. (**a**) Number of yearly volcanic events globally (light blue bars; deep blue color denotes the frequency larger than five per year). Events with volcanic eruption index larger than three are counted and marked by x. (**b**–**d**) Volcanically induced zonal mean net radiative flux (W m^−2^) from December to February (DJF is defined by the year for December) at the top of the model, with the focus on (**b**) period average of 1990–1995 (blue line) and 1996–2001 (gray line), (**c**) year-by-year evolution, and (**d**) the Northern Hemispheric high (60° N–90° N, orange bars) versus middle (15° N–45° N, blue bars) latitudes; green dashed line denotes lower minus higher latitudes, and the black line with dots denotes the volcanic eruptions contribution (%) compared with control simulation. Thick lines and dots over shading denote that the difference had a confidence level of 90%. The Figure was generated using software Grid Analysis and Display System (GrADS) Version 2.1.1.b0 (http://cola.gmu.edu/grads/grads.php).

A sudden and short-lived radiative response to volcanic aerosol was observed as per satellite data and climate modeling; this response differs from a smooth and monotonous change in surface temperature driven by anthropogenic global warming^37^. According to historical records, we investigated how volcanic eruptions, including volcanic sulfur dioxide emissions^24^ and VEI, contributed to the decadal change in the 1990s. As presented in Fig. 1a, the 1991 Mount Pinatubo eruption with the VEI level 6 was rarely observed again over the past half century. We evaluated the decadal distribution of eruptions in the 1990s; a higher volcanic frequency was identified in the earlier than later decade.

The comparison between simulations with and without the 1990s volcanic forcing highlighted the impact of the 1991 eruption of Mount Pinatubo. As shown in Fig. 1b, the (top of the atmosphere) radiation in 1990–1995 has greater differences than 1996–2001 (i.e., anomalous cooling in the lower latitudes and anomalous heating in higher latitudes in the Northern Hemisphere), indicating a volcanically induced reduction in the Equator–Pole radiation contrast. In addition to the 6-year average shown in Fig. 1b, the yearly perspective (Fig. 1c–d) confirms this volcanically-induced reduction in meridional radiation contrast in the early rather than the late 1990s. Without the inclusion of volcanic effects, the meridional radiation contrast at the top of the model is overestimated by approximately 3% in the Northern Hemisphere (Black line with dots, Fig. 1d). With the overestimated temperature change, the global warming assessment is questionable, particularly including tropospheric warming at lower latitudes^19^.

Studies have identified a short-lived tropospheric circulation response to large tropical volcanic eruptions, characterized as mostly the positive AO-like pattern^1,8^. Compared with the nonvolcanic period, the Equator–Pole temperature gradient change caused by the Mount Pinatubo eruption was followed by a stronger polar vortex and an enhanced midlatitude westerly jet stream in the upper troposphere^1,3^. The strengthening and northward shift of the westerly jet stream coincide to the rapid decadal pattern shift in winter in the mid-1990s. Thus, the role of volcanic eruption in modulating and triggering the decadal change that occurred in the 1990s should be determined.

### The North Pacific decadal change in the 1990s

**Figure 2:**
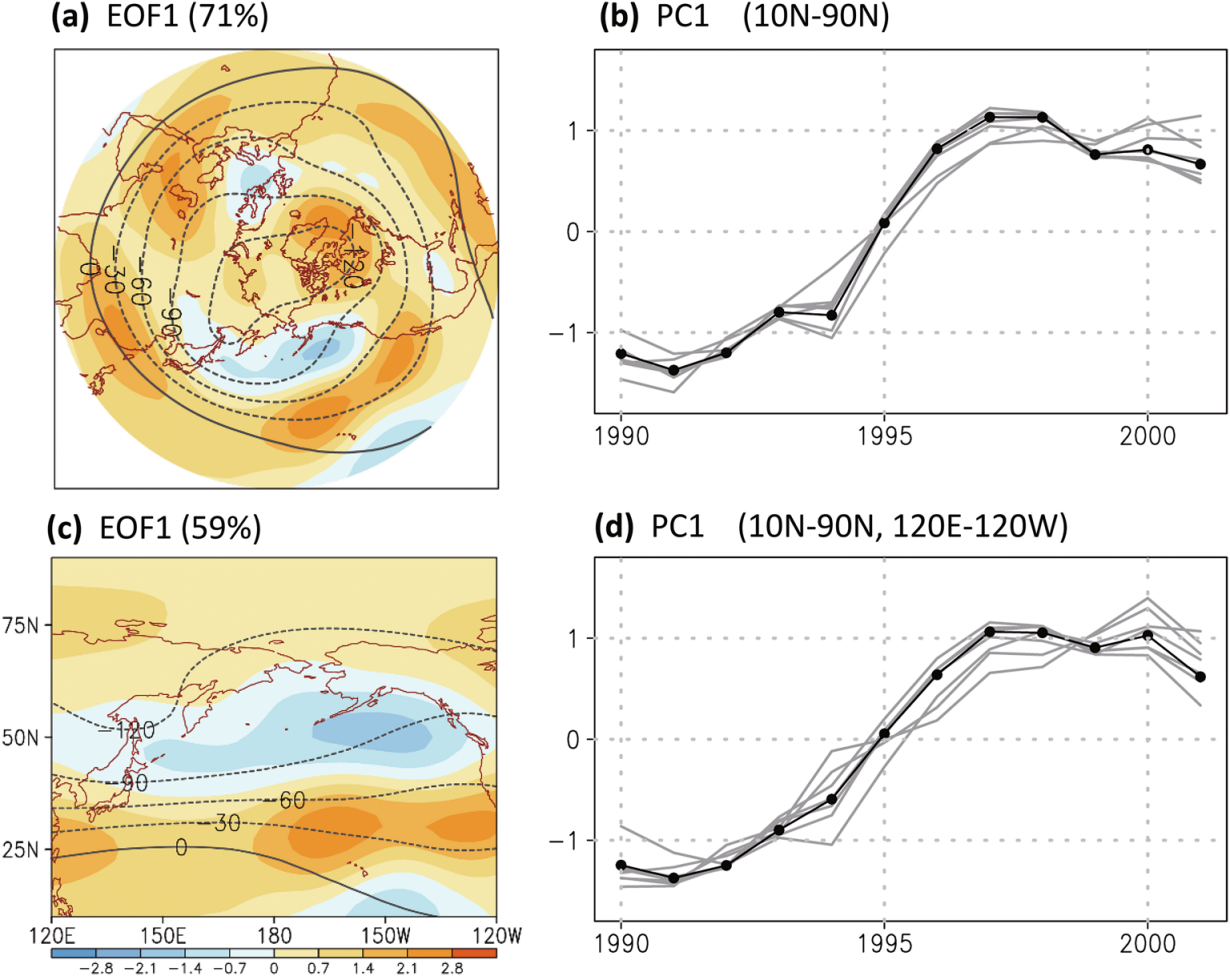
The first mode of empirical orthogonal function analysis (EOF1, shaded) of the 300-hPa streamfunction (unit: 10^6^ m^2^ s^−1^) of the reanalysis ensemble and the corresponding principle component (PC1, black line with dots) in December to February from 1987 to 2004 in the region at (**a**–**b**) 0°–360° E, 10° N–90° N and (**c**–**d**) 120° E–120° W, 10° N–90° N. A 7-year running mean is applied before calculating the EOF analysis. Contour denotes the averaged streamfunction (unit: 10^6^ m^2^ s^−1^) from 1990 to 1995. Gray lines denote the PC1s based on individual reanalysis data. The Figure was generated using software Grid Analysis and Display System (GrADS) Version 2.1.1.b0 (http://cola.gmu.edu/grads/grads.php).

**Figure 3:**
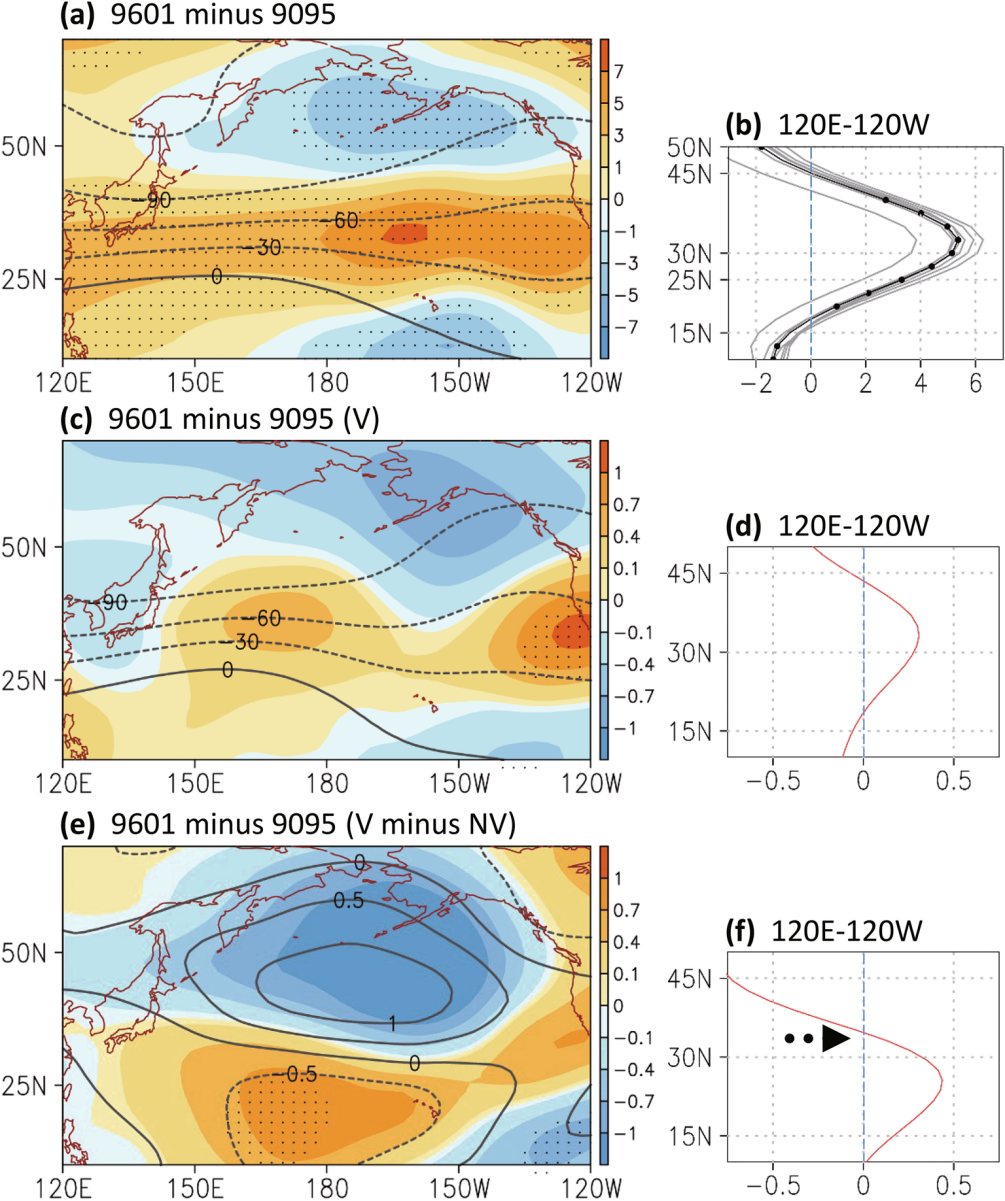
(**a**) The difference (1996–2001 minus 1990–1995) in the streamfunction at 300 hPa (unit: 10^6^ m^2^ s^−1^) of the reanalysis ensemble from December to February. Contour denotes the averaged streamfunction from 1990 to 1995. Dots over shading indicate that the difference in streamfunction had a confidence level of 90%. (**b**) Zonal mean (120° E–120° W) of the 300-hPa streamfunction difference in the seven-reanalysis data (gray; black line with dots for the ensemble mean-based result). (**c**–**d**) Same as (**a**–**b**) except for the decadal change in control simulation with volcanic forcing (V simulation). (**e**–**f**) Same as (**c**–**d**) except for the difference in the modeling decadal change (with minus without volcanic forcing); contour denotes the decadal change without including volcanic forcing (NV simulation). The Figure was generated using software Grid Analysis and Display System (GrADS) Version 2.1.1.b0 (http://cola.gmu.edu/grads/grads.php).

**Figure 4:**
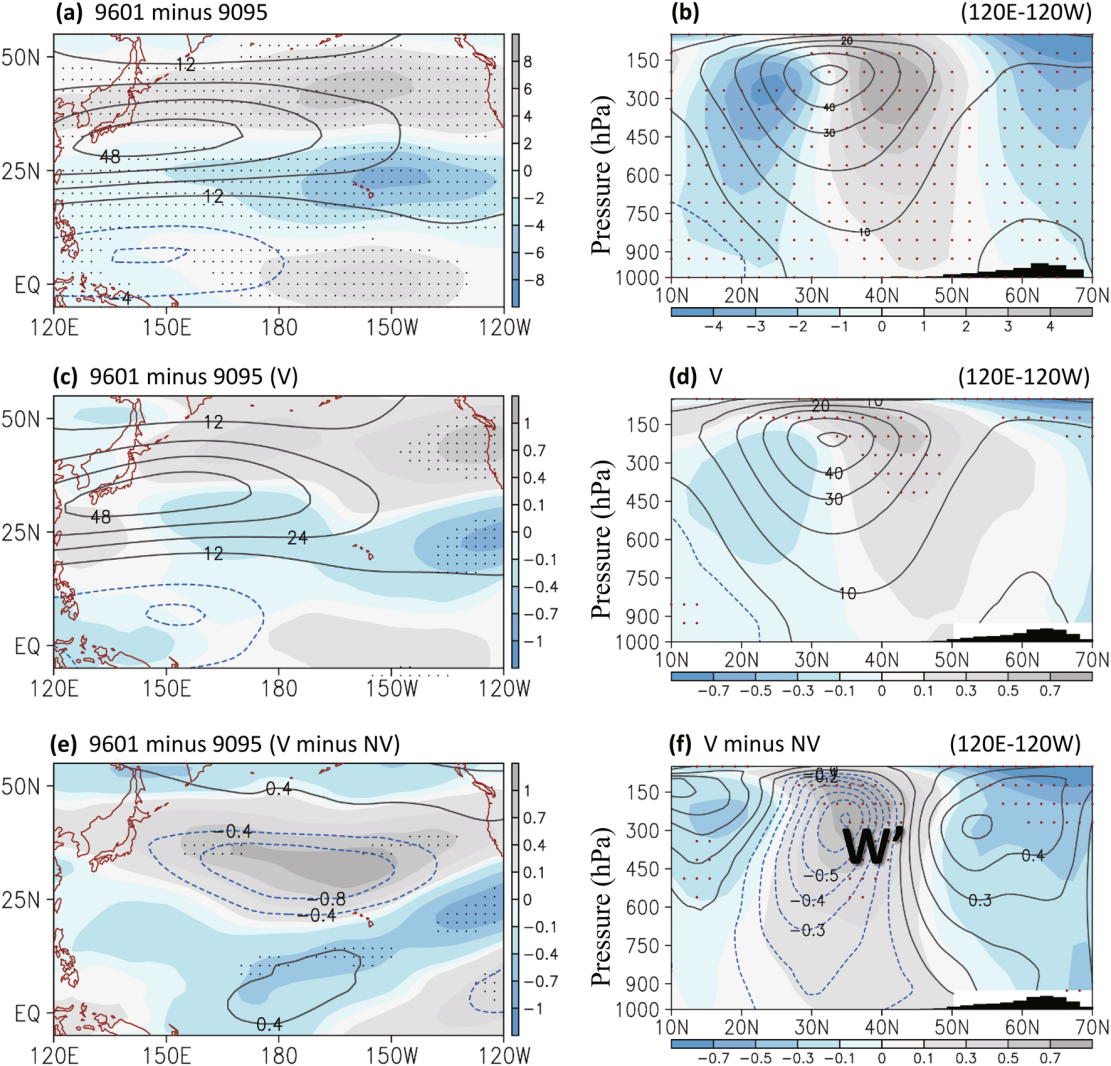
Same as Fig. 3 except for (**a**,**c**,**e**) the averaged zonal winds in 200–500 hPa (m s^−1^) and (**b**,**d**,**f**) the zonal mean (120° E–120° W) of zonal winds in pressure levels. In (**e**–**f**), contour denotes the decadal change without including volcanic forcing (1996–2001 minus 1990–1995 of NV simulation). Dots over shading indicate that the difference in zonal winds had a confidence level of 90%. The Figure was generated using software Grid Analysis and Display System (GrADS) Version 2.1.1.b0 (http://cola.gmu.edu/grads/grads.php).

The decadal climate shift occurred rapidly in winter in the 1990s and resulted in synchronous changes worldwide, including the poleward expansion of an overall dry–wet pattern (which includes the convection over the Indian Ocean–West Pacific warm pool and dry regions in North Africa and West Asia)^23^. In the Northern Hemisphere, a series of abrupt changes occurring in the mid-1990s could be related to the retreat of the Arctic vortex-like circulation in the upper troposphere, displayed as a reduction in cyclonic streamfunction at the border of the vortex pattern (20° N–40° N; Fig. 2a–b; refers to an overall change). Over the North Pacific Ocean, the decadal weakening of the Arctic vortex-like circulation corresponded to the weakening/strengthening of the upper-tropospheric streamfunction in the subtropical/higher latitudes (Fig. 2c–d). Following the EOF analysis, the streamfunction decadal change pattern can be consistently identified in the difference between 1990–1995 and 1996–2001, particularly the peak change around 30° N (Fig. 3a–b). Corresponding to the streamfunction changes, we noted a poleward shift of the midlatitude westerly jet stream (Fig. 4a–b) and the strengthening of the low-level subtropical anticyclone (Fig. 3a of Wu et al.^23^). The synchronous poleward shift of westerly winds vertically (Fig. 4b) implies the efficient coupling of the North Pacific circulation throughout the upper to lower troposphere. We tracked changes in circulation, primarily in streamfunction and zonal winds, to investigate model performance and the atmospheric response to volcanic eruptions in the 1990s.

### Modeling decadal changes with and without volcanic eruptions

**Figure 5:**
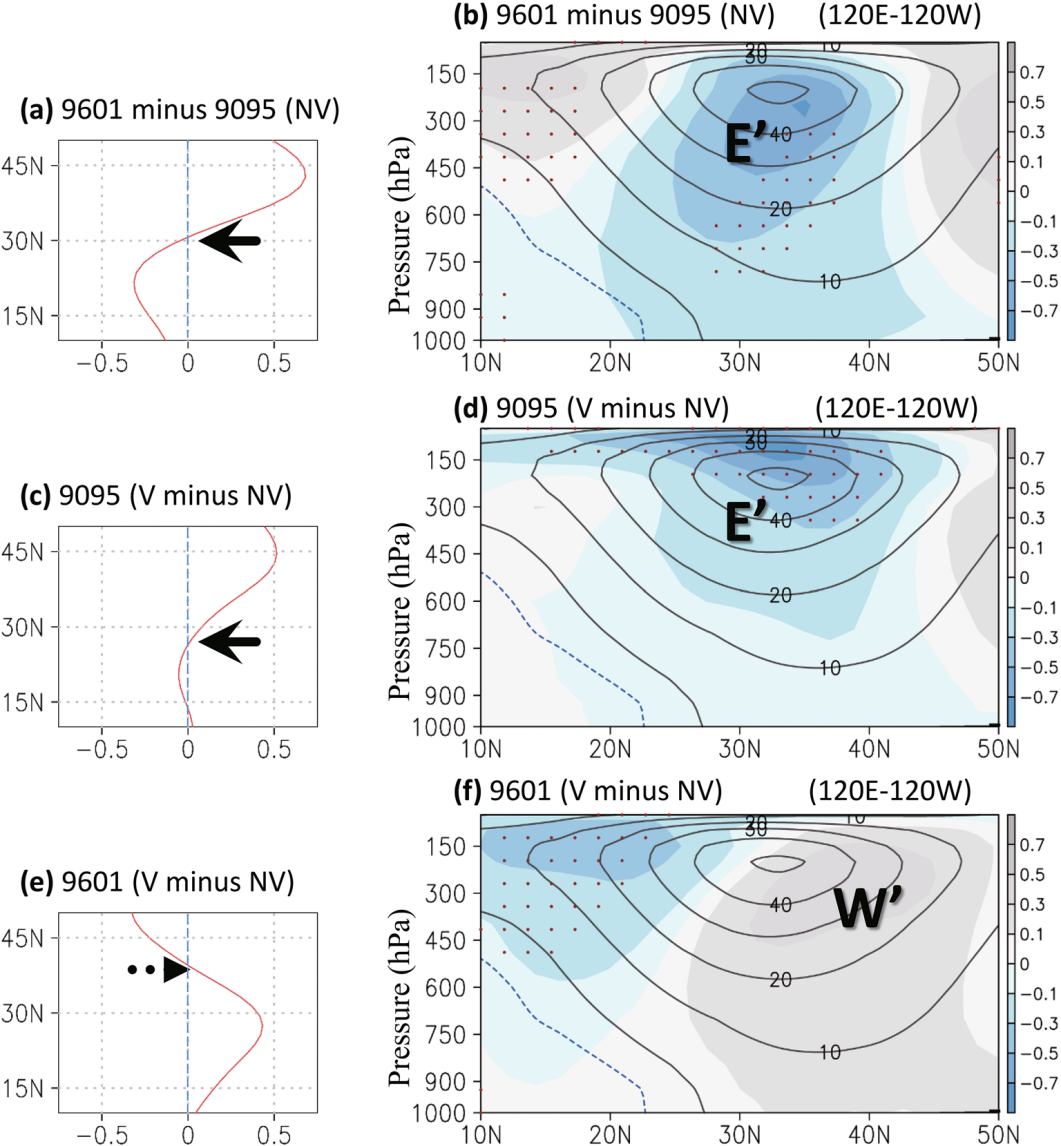
The modeling decadal change (1996–2001 minus 1990–1995) without volcanic forcing from December to February in (a) the zonal mean (120° E–120° W) streamfunction at 300 hPa (unit: 10^6^ m^2^ s^−1^) and (**b**) the zonal mean of zonal winds in pressure levels. Dots over shading indicate that the difference in streamfunction had a confidence level of 90%. The Figure was generated using software Grid Analysis and Display System (GrADS) Version 2.1.1.b0 (http://cola.gmu.edu/grads/grads.php).

We investigated the atmospheric response to the volcanic forcing of the 1990s, focusing on the periods 1996–2001 and 1990–1995 and their difference. When the volcanic effects were considered (V simulation), the model simulated the decadal streamfunction (Fig. 3c–d) and westerly (Fig. 4c–d) changes in the upper troposphere; however, the amplitude of the modeling decadal change was considerably smaller than that observed in reanalysis data. When volcanic aerosol was not included (NV simulation), the decadal change exhibited an opposite pattern over the North Pacific, indicating the strengthening of the subtropical streamfunction (contours in Fig. 3e) and the weakening of the westerly jet stream (Fig. 5b and contours in Fig. 4e–f). This finding suggests the volcanic eruptions contribution to the decadal pattern shift that occurred over the North Pacific in the 1990s. To more clearly observe the volcanically driven decadal change, we compared the decadal change between the control and NV simulation. The results supported the volcanic contribution to the decadal change that occurred in the 1990s, except that the anomalous patterns exhibit a slightly southward shift due to only volcanic aerosol (Fig. 3f; shadings in Fig. 3e and Fig. 4e–f).

When decadal volcanic forcing was excluded from the model, the decadal change occurred gradually in the 1990s (not shown in figure) compared with the rapid change in the mid-1990s. The midlatitude westerly jet stream appeared to weaken when volcanic aerosol was not included (Fig. 5b and contours in Fig. 4e–f). The easterly wind anomaly corresponded to the weakening of the upper tropospheric cyclonic streamfunction over the North Pacific north of approximately 30° N (Fig. 5a). We examined the volcanic impact in the early (1990–1995) and late (1996–2001) decade. The volcanically driven decadal change was noted only in the late 1990s (Fig. 5e–f compared with Figs. 3d and 4d). In the early 1990s (Fig. 5c–d), the circulation response to volcanic aerosol resembled the change in the decadal pattern irrelevant to eruptions (NV simulation, Fig. 5a–b). This may imply a time-lagged characteristic of the volcanic climate impact because a short-lived radiative response to the Pinatubo eruptions was observed during the studied decade.

## Discussion

The accumulation potential of the volcanic climate impact could not be completely confirmed on the basis of only modeling results with decadal forcing. We provide two speculations for the time-lagged response of the North Pacific circulation to the 1990s volcanic eruptions. The short-lived tropospheric response to volcanic aerosol observed in the early 1990s (includes positive AO-like patterns^38,39^), which is an anomalous pattern representing the decadal change under a nonvolcanic condition, can be considered as a precursor process of the rapid decadal change. The inclusion of the volcanic effect may result in a dramatic transition of the decadal state into the next phase. Without the inclusion of the 1990s volcanic aerosol, the decadal change could be performed differently. The midlatitude westerly jet stream weakened gradually over the whole decade; the upper-level streamfunction changed in the opposite direction in the 1990s (Fig. S1). However, whether the volcanically-induced changes caused by such as the Mt. St. Helens eruption in the 1980s, and later the Mt. Pinatubo eruption in the 1990s, would serve as cluster forcing remains an open question. It is worthy investigating further with longer experimental simulations.

Recent modeling studies indicated a volcanically-modulated PDO pattern during the Little Ice Age^12^. The decadal change that occurred in the 1990s may be related to volcanically induced changes in some major climate modes. We examined the PDO and NPGO, which are considered major climate modes over the North Pacific^40–42^. Because studies have emphasized the volcanically induced AO-like circulation change, we examined whether the AO-related pattern undergoes a marked change across the 1990s. We compared the power spectrum of focused climate indices between 1951–1990 and 1981–2020; the 1990s condition could be ruled out in the spectral analysis of the period from 1951 to 1990. Spectral signals in the semi-decade (approximately between 3- and 7-year periods) are strengthened in 1981–2020 compared with that in 1951–1990 (Fig. S2a–c), whereas the decadal signal is weakened (particularly in AO and PDO). Next, we examined decadal changes (1996–2001 subtracted by 1990–1995) in sea level pressure and lower tropospheric circulation that were regressed based on the individual climate index. As presented in Fig. S2d–f, AO- and PDO-related circulation changes represented a cyclonic gyre in the lower troposphere over the northwestern Pacific, whereas NPGO-related changes represented a dipole gyre pattern over the region. These anomalous circulation patterns corresponded to the strengthened midlatitude westerly flow in 30° N–40° N, which was similar to the response to the Mount Pinatubo eruption.

Recent studies have emphasized the absence of multidecadal oscillation in climate model simulations^43,44^. The model bias could be partly related to the unrealistic representation of the volcanic impact. However, due to a limited understanding of the accumulation potential, whether the volcanic contribution to long-range climate perturbations would compete with other climatic forcings remains unclear. Distinguishing volcanically induced changes, particularly in terms of the extent to which they can serve as a modulator of decadal changes, is crucial and can help understanding the origin of climate variability and model performance for the climate assessment.

## Conclusion

Because volcanically induced changes pose a major challenge for decadal predictability and climate assessment, this study explored how the 1990s decadal climate pattern shifts can be volcanically modulated. We investigated simulated decadal changes in the 1990s with and without volcanic aerosols and determined the atmospheric change that occurred in winter over the North Pacific Ocean. The contrast in the changing dominance pattern of climate may be closely related to the short-lived radiative impact driven by the 1991 Mount Pinatubo eruption. With the volcanic forcing of the 1990s, the decadal change with a synchronous poleward shift of westerly winds is well represented throughout the upper to lower troposphere over the North Pacific Ocean. This change in zonal wind corresponds to the retreat of the Arctic vortex-like circulation in the upper troposphere. When volcanic aerosols were excluded from the decade, the decadal change exhibited an opposite pattern (i.e., a strengthened subtropical streamfunction and a weakened westerly jet stream), suggesting a substantial effect of large tropical volcanic eruptions on the 1990s decadal pattern shift.

Volcanically driven pattern changes can be clearly identified in the mid-to-late 1990s than in the early 1990s, implying a time-lagged volcanic contribution to the decadal change. Compared with a relatively gradual change observed in the nonvolcanic decade, the inclusion of volcanic effects in the model accelerates the decadal change. Regarding the volcanic contribution to decadal dynamics, volcanically induced changes might be considered a modulator of climate variability.

## Supplementary Information

Supplementary Information.

## Data Availability

All the datasets used in this study are publicly available. The reanalysis data can be obtained from the websites of NASA (MERRA2), ECMWF (ERA5 and ERA20C), NOAA (20CRv3), NCEP (NCEP R1 and CFSR), and JRA55. The volcanic explosivity index is available the Global Volcanism Program of National Museum of Natural History (https://volcano.si.edu/). We obtained AO and PDO indices from NOAA’s website (https://psl.noaa.gov/) and NPGO index from the website (http://www.o3d.org/npgo/npgo.php). The volcanic experiments conducted using NorESM are included in Bethke, et al.^34^ and are available in the corresponding data archives^35^ (https://doi.org/10.11582/2017.00006). All the analyzed results are available from the corresponding author on reasonable request.
