# Supplementary material for: Volcanic contribution to the 1990s North Pacific climate shift in winter
Source: Sci Rep. 2023 Apr 6;13:5672. doi: 10.1038/s41598-023-32956-z (PMC10079832; doi:10.1038/s41598-023-32956-z)
Supplement: Supplementary file 1 — Supplementary Information. [file 41598_2023_32956_MOESM1_ESM.doc]

**Supplementary**


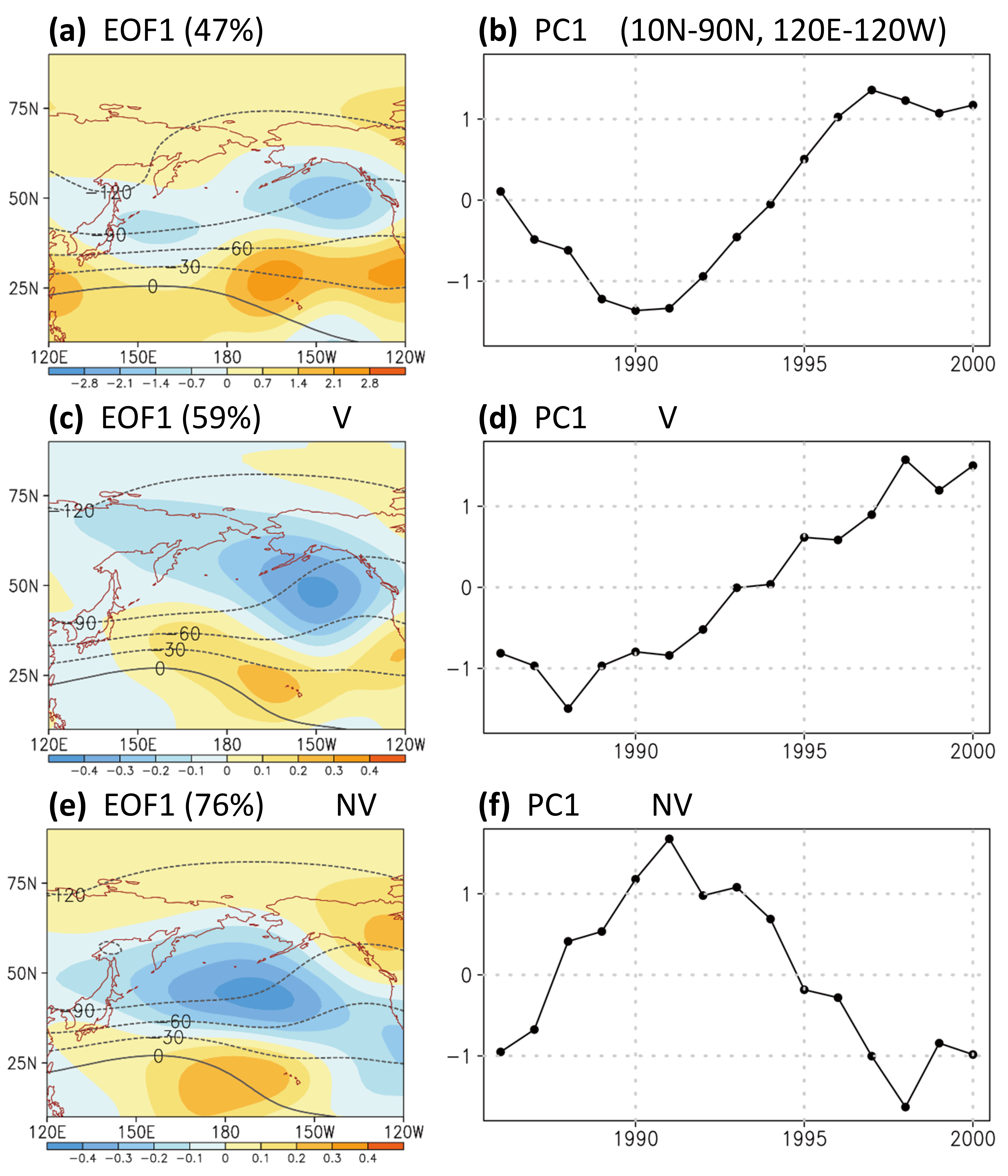


**Figure S1 |** Similar to Fig. 2c–d, but with 7 additional years before 1990 (represented by a 7-year running mean from 1986 to 1990) for (a–b) 7-reanalysis ensemble, (c–d) V simulation, and (e–f) NV simulation.


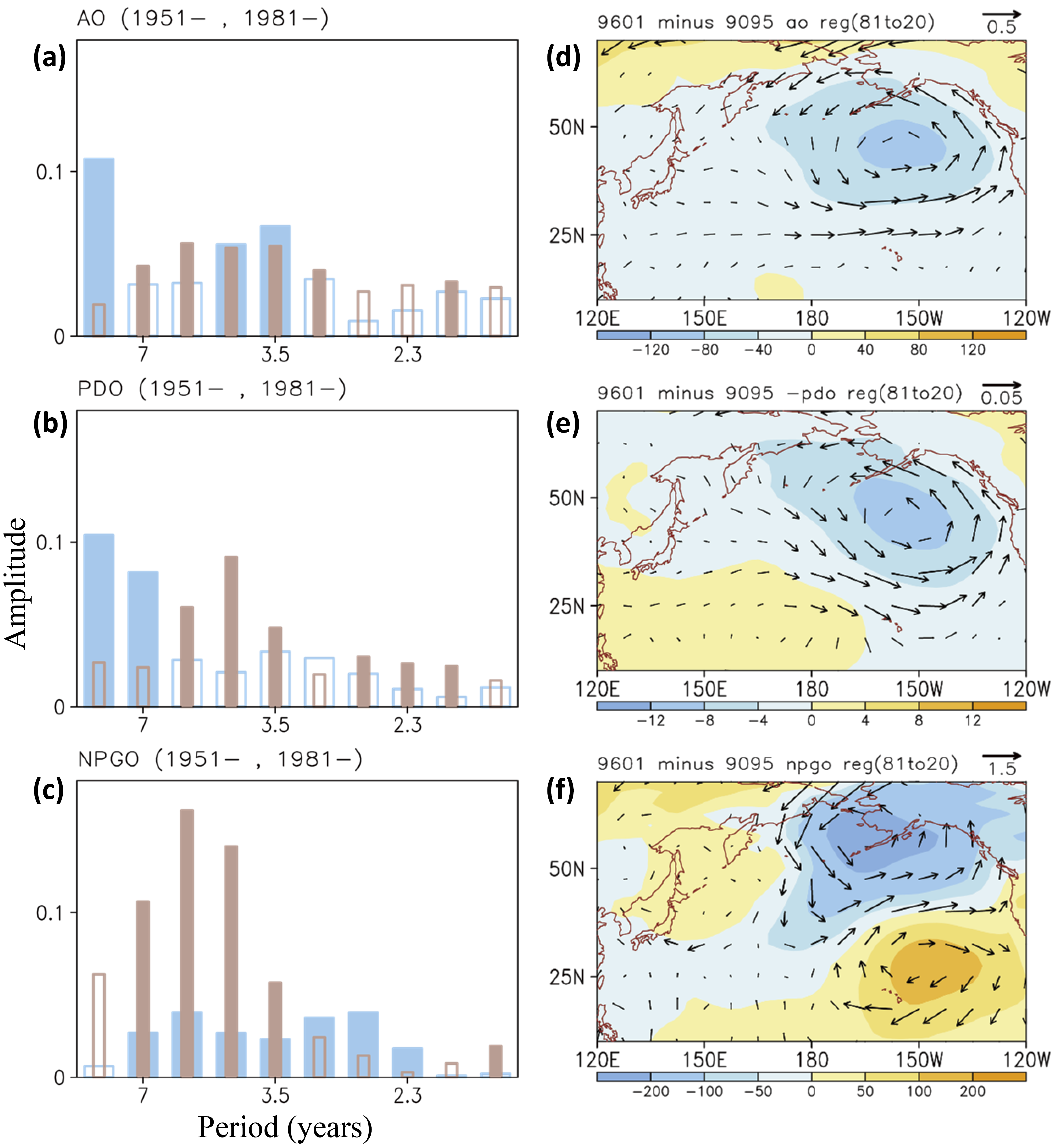


**Figure S2 |** (a–c) Spectral analysis of AO, PDO, and NPGO indices from December to February from 1951 to 1990 (blue) and from 1981 to 2020 (brown). The filled bars denote the corresponding values at a confidence level of 95%. (d–f) The difference (1996–2001 minus 1990–1995) in the regressed sea level pressure (unit: hPa) and horizontal winds at 925 hPa (unit: m s^−1^); the seasonal mean dynamical fields (from NCEP R1 dataset) are regressed onto the AO, PDO, and NPGO index separately in the period 1981–2020.
